# Respiratory syncytial virus reinfections among infants and young children in the United States, 2011–2019

**DOI:** 10.1371/journal.pone.0281555

**Published:** 2023-02-16

**Authors:** Sabina O. Nduaguba, Phuong T. Tran, Yoonyoung Choi, Almut G. Winterstein

**Affiliations:** 1 Department of Pharmaceutical Systems and Policy, College of Pharmacy, West Virginia University, Morgantown, WV, United States of America; 2 West Virginia University Cancer Institute, Morgantown, WV, United States of America; 3 Department of Pharmaceutical Outcomes and Policy, College of Pharmacy, University of Florida, Gainesville, FL, United States of America; 4 Center for Drug Evaluation and Safety, University of Florida, Gainesville, FL, United States of America; 5 Faculty of Pharmacy, HUTECH University, Ho Chi Minh City, Vietnam; 6 Center for Observational and Real-World Evidence, Merck & Co., Inc., Kenilworth, NJ, United States of America; 7 Department of Epidemiology, College of Medicine and College of Public Health and Health Professions, University of Florida, Gainesville, FL, United States of America; Nazarbayev University School of Medicine, PAKISTAN

## Abstract

**Background:**

Although respiratory syncytial virus (RSV) immunoprophylaxis is recommended for high-risk infants, the American Academy of Pediatrics (AAP) recommends against immunoprophylaxis in the same season following a breakthrough hospitalization due to limited risk for a second hospitalization. Evidence in support of this recommendation is limited. We estimated population-based re-infection rates from 2011–2019 in children <5 years since RSV risk remains relatively high in this age group.

**Materials and methods:**

Using claims data from private insurance enrollees, we established cohorts of children <5 years who were followed to ascertain annual (July 1-June 30) and seasonal (November 1- February 28/29) RSV recurrence estimates. Unique RSV episodes included inpatient encounters with RSV diagnosis ≥30 days apart, and outpatient encounters ≥30 days apart from each other as well as from inpatient encounters. The risk of annual and seasonal re-infection was calculated as the proportion of children with a subsequent RSV episode in the same RSV year/season.

**Results:**

Over the 8 assessed seasons/years (N = 6,705,979) and across all age groups annual inpatient and outpatient infection rates were 0.14% and 1.29%, respectively. Among children with a first infection, annual inpatient and outpatient re-infection rates were 0.25% (95% confidence interval (CI) = 0.22–0.28) and 3.44% (95% CI = 3.33–3.56), respectively. Both infection and re-infection rates declined with age.

**Conclusion:**

While medically-attended re-infections contributed numerically only a fraction of the total RSV infections, re-infections among those with previous infection in the same season were of similar magnitude as the general infection risk, suggesting that a previous infection may not attenuate the risk for a re-infection.

## Introduction

Respiratory syncytial virus (RSV) is the leading cause of lower respiratory tract infections among infants and young children [1–4] with most infections occurring during the winter season [5]. Based on small prospective surveillance data, most children have been infected by the virus by age 2 years with 36–42% experiencing at least two infections [6, 7]. Previous RSV infections do not provide long term immunity but re-infections tend to be milder than the initial episode [7–9]. Typical immune response involves the production of neutralizing antibodies in response to an infection but antibody titers drop soon after the infection episode [9]. However, mean serum neutralizing antibody titers were sustained for a longer period following successive infections, which may explain why re-infections are less severe [9].

Previous studies on the epidemiology of RSV re-infection have typically defined re-infection as a new infection occurring during the next RSV season, with a few exceptions [7, 9]. In a study of 726 Japanese children followed over eight seasons, there was only one case of re-infection during the same season occurring 10 days after the initial infection [10]. Further, in a study of 1,287 children with congenital heart disease two years or younger and followed for 150 days, 97 RSV hospitalizations were reported with five children having a re-infection [11]. In a different study of 429 preterm infants less than six months of age, 40 RSV infections were recorded with no re-infections by age 1 [12]. These studies taken together imply that, although not common, re-infections are possible within the same season. However, these findings are based on small and specialized cohorts, which do not provide stable estimates of re-infection rates.

Age is an important risk factor for RSV infection [13]. Accordingly, RSV immunoprophylaxis, which provides passive immunity, is approved and recommended in the US for high-risk infants mainly during the first year of life. Based on the available evidence from prospective studies, the American Academy of Pediatrics recommends against continuing immunoprophylaxis in the same season following an RSV infection [14, 15]. However, because the available evidence on RSV reinfection risk is limited and because RSV infection risk extends beyond the first year of life [16, 17], the patient-specific risk and public health impact of RSV reinfection in the general population of children in the United States is unclear. We estimated the rate of medically-attended re-infections within each RSV year and season in a national cohort of children from 2011 to 2019.

## Materials and methods

We established cohorts of commercially-insured children aged 0 to 4 years using 2011–2019 data from the IBM MarketScan Commercial Claims and Encounters Database. MarketScan provides medical encounter and outpatient pharmacy dispensing detail for a national sample of persons with employer-sponsored health insurance, their spouses, and dependents, covering over 130 million lives between 2011 and 2019. To maximize the capture of all health services received, we excluded children in capitated plans. Medical encounter information includes diagnoses and procedures and related service dates. The study was exempted from review by the University of Florida Institutional Review Board due to use of deidentified data.

We conducted assessments per year (July to June). We included children 0–4 years as of July of each year between 2011 and 2019 and required continuous enrollment over the entire year to ensure capture of the index RSV infection in each year and to facilitate estimation of incidence rates that spanned the entire observation period.

We identified RSV-related admissions and ambulatory encounters using diagnosis codes based on the International Classification of Diseases, Ninth Revision, Clinical Modification (ICD-9-CM) and the International Classification of Diseases, Tenth Revision, Clinical Modification (ICD-10-CM) (ICD-9 codes: 4801, 46611, 0796; ICD-10 codes: J121, J210, J205, B974). In an attempt to focus on community-acquired RSV, we further excluded all encounters with a birth/delivery diagnostic code among children 0 years and adjusted the observation period to begin the day after a birth hospitalization so that follow-up could begin in ambulatory care.

Because an RSV episode may require multiple medical encounters before resolution, we applied a case identification algorithm to define unique episodes within each RSV year. We first identified unique inpatient episodes from the selection of RSV-related inpatient encounters by requiring at least 30 days between adjacent encounter claims. This 30-day requirement served as a washout period to determine the end of an episode. Next, we identified unique outpatient episodes by first removing outpatient RSV-related encounters within 30 days of identified inpatient episodes and then applying the 30-day washout rule to the remaining outpatient encounters. We used this hierarchical approach of identifying medically-attended inpatient episodes before outpatient episodes to ensure comprehensive capture of all unique inpatient episodes, which we expected to be more severe. An episode of RSV re-infection was defined as a unique medically-attended episode occurring after the medically-attended index episode in each year.

### Analysis

We estimated the annual incidence of RSV infection as the number of children with at least one RSV infection in each year divided by the total number of children enrolled/at risk in that year. The annual RSV re-infection rate was then estimated as the number of children with at least one re-infection divided by the number of children with an initial (index) RSV episode in that year, agnostic of whether the initial episode occurred in the inpatient or outpatient setting. The annual estimates were determined separately for the inpatient and outpatient settings. The degree of uncertainty in the estimates was calculated as the 95% confidence intervals for proportions using the Wald method. Negative lower confidence limits were truncated to zero.

We conducted additional analyses to derive seasonal estimates including core RSV seasons defined as November 1 to February 28 (or 29 for leap years). Although RSV seasonality varies by region, these months are typically included in the RSV season across years and regions in the United States [18]. Two sets of analyses were conducted: 1) including children 0–4 years as of November for core season assessments of each year between 2011 and 2019 and requiring continuous enrollment over the entire core RSV season; and 2) including children 0–4 years as of July but only counting RSV infections and re-infections during November to February. This was done to allow for a direct comparison of annual and seasonal estimates within the same cohort since the age entry criteria at different calendar months result in a younger cohort of children for seasonal estimates.

To assess the effect of different assumptions about the maximum time a RSV episode resolves, we also repeated the analyses of annual rates requiring a 15-day and a 45-day washout period. We also reported annual incidence estimates for RSV-associated lower respiratory tract re-infections (RSV-LRTI) following an index RSV-LRTI. RSV-LRTI was defined as any diagnostic code for pneumonia (ICD-9 code: 480.1; ICD-10 code: J12.1), bronchitis (ICD-10 code: J20.5), or bronchiolitis (ICD-9 code: 466.11; ICD-10 code: J21.0) due to RSV, or a primary code for pneumonia (ICD-9 code: 480, 480.9, 484.8, 486; ICD-10 code: J12, J12.9, J18), bronchitis (ICD-10 code: J20, J20.9), or bronchiolitis (ICD-9 code: 466.1; ICD-10 code: J21, J21.9) with unspecified pathogen plus a secondary code for RSV (ICD-9 code: 079.6; ICD-10 code: B97.4).

All analyses were conducted using SAS 9.04.01.M6.

## Results

**Fig 1:**
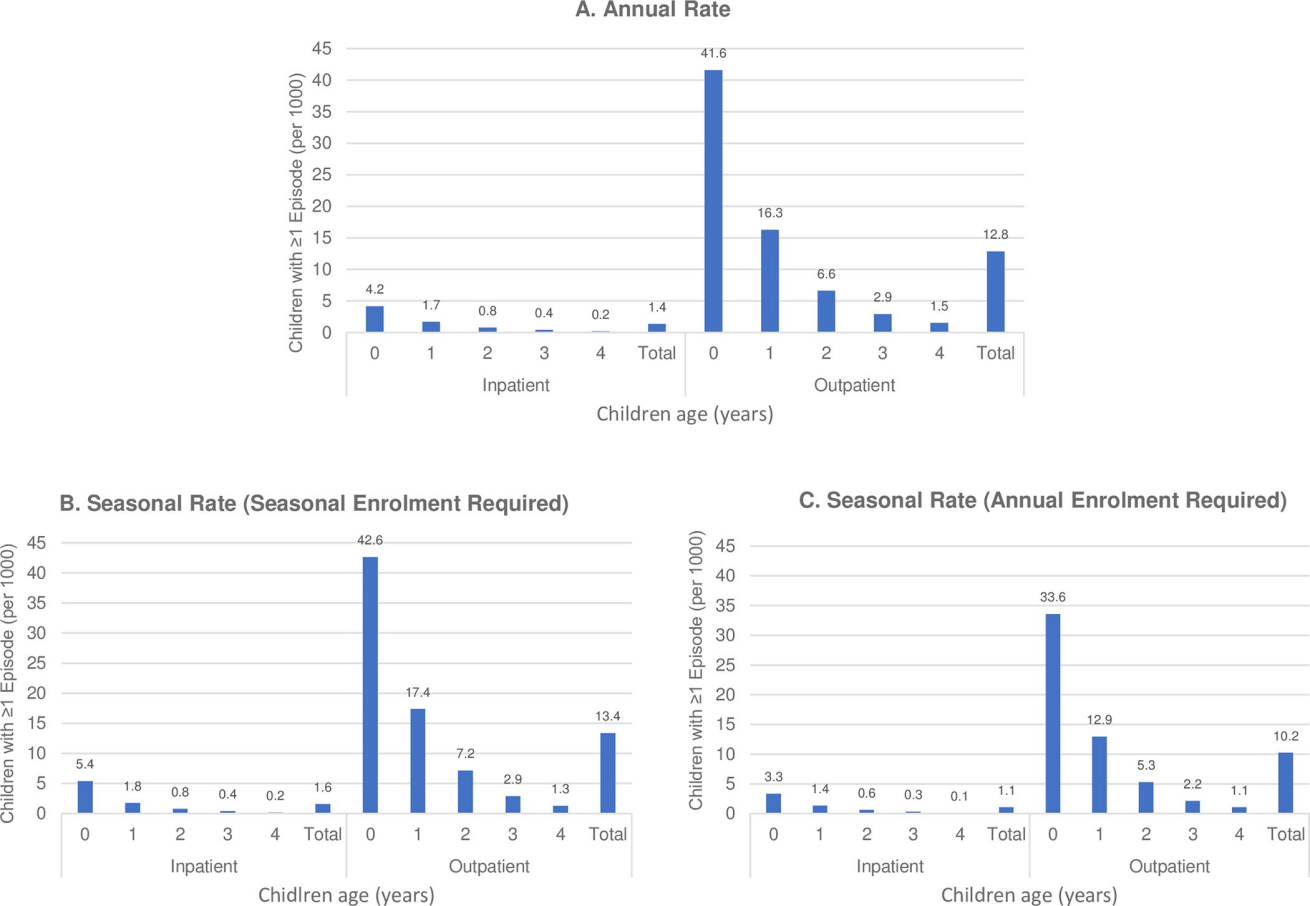
Respiratory syncytial virus infection rate involving inpatient or outpatient care among commercially-insured children 0–4 years, 2011–2019. **A.** annual rate (children age as of July 1), **B.** seasonal rate (constrained to November to February, children age as of November 1), **C.** seasonal rate estimated in the annual cohort (children age as of July 1).

There were 6,705,979 children included in the study ranging from 687,763 to 1,110,528, depending on the year. **Fig 1** shows the total annual and seasonal infection rates pooled across calendar years 2011–2019 stratified by age and overall. **S1 Table** shows annual rates further stratified by RSV year. Pooled across years, annual infection rates resulting in inpatient encounters were 4.2 per 1,000 children less than 1 year old at the beginning of July, and 41.6 in the outpatient setting. Infection rates declined with increasing age to 0.2 in the inpatient and 1.5 per 1,000 in the outpatient setting for children 4 years old. Similarly, seasonal infection rates per 1,000 children declined from 5.4 in the inpatient setting and 42.6 in the outpatient setting for children less than 1 year old at the beginning of the season to 0.2 and 1.3, respectively, for children 4 years old. Seasonal rates for the annual cohort using age thresholds at the beginning of July (for direct comparison with annual rates) were 3.3 for inpatient and 33.6 for outpatient infections per 1,000 children age 0. Assessment during the four core season months captured more than three quarters of all infections.

**Fig 2:**
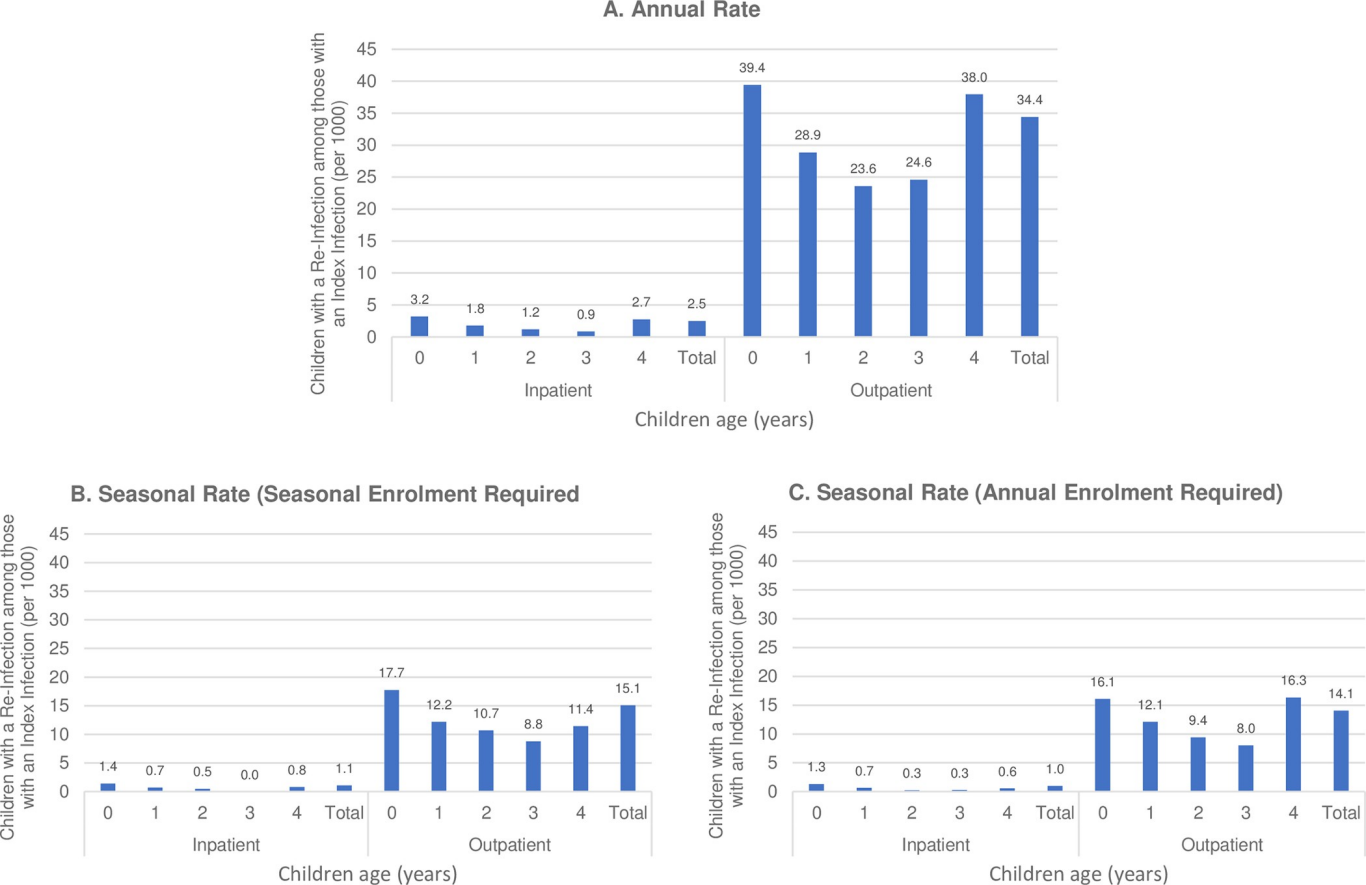
Respiratory Syncytial Virus (RSV) re-infection rate involving inpatient or outpatient care among commercially-insured children 0–4 years with an index inpatient or outpatient episode, 2011–2019. **A.** annual rate (children age as of July 1), **B.** seasonal rate (constrained to November to February, children age as of November 1), **C.** seasonal rate estimated in the annual cohort (children age as of July 1).

**Fig 2** shows annual and seasonal re-infection rates pooled across calendars years 2011–2019 stratified by age and overall. Annual inpatient and outpatient re-infection rates per 1,000 children 0 to 4 years of age with an initial RSV episode in the same year were 2.5 and 34.4, respectively. Annual inpatient re-infection rates declined from 3.2 to 0.9 from age 0 to age 3 but increased again to 2.7 among children age 4 and annual outpatient re-infection rates were similar with a trough at 23.6 infections per 1,000 2-year olds. Comparing seasonal rates among the same annual cohort with age thresholds for cohort entry defined in July, seasonal assessments captured less than half the infections that occurred over the year. Seasonal estimates over November to February for both inpatient and outpatient re-infection rates were lower at 1.1 and 15.1 infections per 1,000 children age 0 to 4, respectively. Seasonal age-specific re-infection rates ranged from 0.0 per 1,000 3 year olds to 1.4 per 1,000 per 0 year olds and outpatient re-infection rates ranging from 8.8 per 1,000 3 year olds to 17.7 per 0 year olds. **Tables 1 and 2**, respectively, detail the total number of children with an index RSV episode, the total number of inpatient (Table 1) and outpatient (Table 2) re-infections, the number of children with at least one re-infection, and re-infection rates for each assessed RSV-year. Overall, there were 2,555 children with an index episode across all eight years with 7 and 97 children having one or more inpatient or outpatient re-infections, respectively. Notably, there were zero inpatient re-infections in one, two, four, and four of the eight years among children 1, 2, 3, and 4 years old, respectively.

**Table 1:** Annual inpatient respiratory syncytial virus re-infection rate among commercially-insured children 0–4 years with an index inpatient or outpatient episode in the same year, 2011–2019 ^a^.

	Children with Index Episode in either Inpatient or Outpatient Setting (N)					
	Number of Inpatient Re-infections					
	Children with ≥1 Inpatient Re-infection (N)					
	Inpatient Re-infection Rate, % (95% Confidence Interval)					
	Overall	0 Years	1 Year	2 Years	3 Years	4 Years
2011–2012	14,533	8,181	3,515	1,555	774	508
	49	36	9	3	1	0
	49	36	9	3	1	0
	0.34 (0.24–0.43)	0.44 (0.30–0.58)	0.26 (0.09–0.42)	0.19 (0.00–0.41)^b^	0.13 (0.00–0.38)^b^	0.00 (0.00–0.00)
2012–2013	12,898	7,342	3,190	1,340	647	379
	29	16	11	0	1	1
	29	16	11	0	1	1
	0.22 (0.14–0.31)	0.22 (0.11–0.32)	0.34 (0.14–0.55)	0.00 (0.00–0.00)	0.15 (0.00–0.46)^b^	0.26 (0.00–0.78)^b^
2013–2014	12,033	7,051	2,873	1,216	602	291
	32	26	4	2	0	0
	31	25	4	2	0	0
	0.26 (0.17–0.35)	0.35 (0.22–0.49)	0.14 (0.00–0.28)	0.16 (0.00–0.39)^b^	0.00 (0.00–0.00)	0.00 (0.00–0.00)
2014–2015	11,317	6,522	2,747	1,231	524	293
	26	22	0	1	1	2
	24	20	0	1	1	2
	0.21 (0.13–0.30)	0.31 (0.17–0.44)	0.00 (0.00–0.00)	0.08 (0.00–0.24)^b^	0.19 (0.00–0.56)^b^	0.68 (0.00–1.63)^b^
2015–2016	11,211	6,306	2,840	1,216	568	281
	28	21	5	1	1	0
	28	21	5	1	1	0
	0.25 (0.16–0.34)	0.33 (0.19–0.48)	0.18 (0.02–0.33)	0.08 (0.00–0.24)^b^	0.18 (0.00–0.52)^b^	0.00 (0.00–0.00)
2016–2017	10,883	6,396	2,637	1,122	484	244
	22	19	1	2	0	0
	21	18	1	2	0	0
	0.19 (0.11–0.28)	0.28 (0.15–0.41)	0.04 (0.00–0.11)^b^	0.18 (0.00–0.43)^b^	0.00 (0.00–0.00)	0.00 (0.00–0.00)
2017–2018	10,296	6,044	2,434	1,060	492	266
	29	17	6	3	0	3
	28	17	6	3	0	2
	0.27 (0.17–0.37)	0.28 (0.15–0.41)	0.25 (0.05–0.44)	0.28 (0.00–0.60)^b^	0.00 (0.00–0.00)	0.75 (0.00–1.79)^b^
2018–2019	12,098	7,063	2,863	1,298	581	293
	30	22	6	0	0	2
	29	22	5	0	0	2
	0.24 (0.15–0.33)	0.31 (0.18–0.44)	0.17 (0.02–0.33)	0.00 (0.00–0.00)	0.00 (0.00–0.00)	0.68 (0.00–1.63)^b^
Total	95,269	54,905	23,099	10,038	4,672	2,555
	245	179	42	12	4	8
	239	175	41	12	4	7
	0.25 (0.22–0.28)	0.32 (0.27–0.37)	0.18 (0.12–0.23)	0.12 (0.05–0.19)	0.09 (0.00–0.17)	0.27 (0.07–0.48)

^a^Index episode may occur in either the inpatient or outpatient setting

^b^Negative 95% confidence limit truncated to 0.00%

**Table 2:** Annual outpatient respiratory syncytial virus re-infection rate among commercially-insured children 0–4 years with an index inpatient or outpatient episode in the same year, 2011–2019 ^a^.

	Children with Index Episode in either Inpatient or Outpatient Setting (N)					
	Number of Outpatient Re-infections					
	Children with ≥1 Outpatient Re-infection (N)					
	Outpatient Re-infection Rate (95% Confidence Interval)					
	Overall	0 Years	1 Year	2 Years	3 Years	4 Years
2011–2012	14,533	8,181	3,515	1,555	774	508
	766	490	145	62	38	31
	645	419	121	49	31	25
	4.44 (4.10–4.77)	5.12 (4.64–5.60)	3.44 (2.84–4.05)	3.15 (2.28–4.02)	4.01 (2.62–5.39)	4.92 (3.04–6.80)
2012–2013	12,898	7,342	3,190	1,340	647	379
	590	400	114	39	14	23
	503	345	93	34	14	17
	3.90 (3.57–4.23)	4.70 (4.21–5.18)	2.92 (2.33–3.50)	2.54 (1.70–3.38)	2.16 (1.04–3.28)	4.49 (2.40–6.57)
2013–2014	12,033	7,051	2,873	1,216	602	291
	597	390	131	34	20	22
	513	329	116	33	18	17
	4.26 (3.90–4.62)	4.67 (4.17–5.16)	4.04 (3.32–4.76)	2.71 (1.80–3.63)	2.99 (1.63–4.35)	5.84 (3.15–8.54)
2014–2015	11,317	6,522	2,747	1,231	524	293
	509	330	98	47	22	12
	433	283	85	41	14	10
	3.83 (3.47–4.18)	4.34 (3.84–4.83)	3.09 (2.45–3.74)	3.33 (2.33–4.33)	2.67 (1.29–4.05)	3.41 (1.33–5.49)
2015–2016	11,211	6,306	2,840	1,216	568	281
	358	235	78	25	13	7
	313	206	70	22	9	6
	2.79 (2.49–3.10)	3.27 (2.83–3.71)	2.46 (1.89–3.04)	1.81 (1.06–2.56)	1.58 (0.56–2.61)	2.14 (0.45–3.83)
2016–2017	10,883	6,396	2,637	1,122	484	244
	305	192	71	29	7	6
	262	165	58	26	7	6
	2.41 (2.12–2.70)	2.58 (2.19–2.97)	2.20 (1.64–2.76)	2.32 (1.44–3.20)	1.45 (0.38–2.51)	2.46 (0.52–4.40)
2017–2018	10,296	6,044	2,434	1,060	492	266
	347	224	75	22	12	14
	298	194	66	17	9	12
	2.89 (2.57–3.22)	3.21 (2.77–3.65)	2.71 (2.07–3.36)	1.60 (0.85–2.36)	1.83 (0.65–3.01)	4.51 (2.02–7.01)
2018–2019	12,098	7,063	2,863	1,298	581	293
	378	252	77	22	19	8
	313	223	58	15	13	4
	2.59 (2.30–2.87)	3.16 (2.75–3.57)	2.03 (1.51–2.54)	1.16 (0.57–1.74)	2.24 (1.03–3.44)	1.37 (0.04–2.69)
Total	95,269	54,905	23,099	10,038	4,672	2,555
	3,850	2,513	789	280	145	123
	3,280	2,164	667	237	115	97
	3.44 (3.33–3.56)	3.94 (3.78–4.10)	2.89 (2.67–3.10)	2.36 (2.06–2.66)	2.46 (2.02–2.91)	3.80 (3.06–4.54)

^a^Index episode may occur in either the inpatient or outpatient setting

Sensitivity analyses requiring a 15-day washout showed slightly higher inpatient (3.0 versus 2.5 when pooled across years and age groups) and outpatient annual re-infection rates (36.2 versus 34.4) compared to the 30-day washout period (**S1 Fig, S2 and S3 Tables**). Sensitivity analyses requiring 45-day washouts showed slightly lower annual re-infection rates (**S1 Fig, S4 and S5 Tables**). In the sensitivity analyses including only RSV-related LRTI, inpatient and outpatient re-infection rates were lower among those who had at least one RSV-related LRTI although the difference was minor when compared to RSV infections at any site (**S1 Fig, S6 and S7 Tables**).

## Discussion

In this study based on over 6 million commercially-insured children 4 years or younger, we sought to determine how frequently RSV re-infections occurred within the same RSV year or season. Similar to other studies, a trend of declining rates with increasing age was observed [16, 19]. With RSV infection risk heavily relying on age, especially among infants less than 1 year of age [13], comparison of incidence estimates to other studies need to consider age-related study entry criteria in addition to case ascertainment methods (e.g., prospective assessments versus reliance on need for medical care) [20–22].

Inpatient and outpatient RSV infection rates were 1.4 and 12.9 per 1,000 children, respectively, which is comparable to global estimates for high income countries [23]. Among children who had an index RSV episode, annual inpatient and outpatient re-infection rates were 2.5 and 34.4 per 1,000 children across all age groups, respectively, which compares to observed infection rates. Likewise, reinfections among children less than one year of age were 3.2 and 39.4 for inpatient and outpatient encounters, compared to RSV infection rates for this age group of 4.3 and 41.7, respectively. In other words, the reinfection risk among those with previous infection in the same season appears to be of similar magnitude or slightly higher than the baseline risk for infection. Because the population at risk in our estimate of re-infection rates were children with an initial infection, the contribution of reinfection to the overall burden of medically-attended RSV disease is indeed marginal. However, the individual risk for a first infection or a second infection following a first infection are of similar magnitude.

Current AAP recommendations include not continuing “monthly immunoprophylaxis among children who experience a breakthrough RSV hospitalization due to low likelihood of a second RSV hospitalization [14, 15].” Although this reasoning may deserve reconsideration given our finding of similar reinfection risk as baseline infection risk, the re-infection rates seen in our study might not reach thresholds that are typically considered when defining high-risk populations that are recommended for prophylaxis. Our study of RSV incidence estimated inpatient rates of 116–396 per 100,000 children months among high-risk children with extreme prematurity, chronic lung disease, congenital heart disease, Down’s syndrome, cystic fibrosis, neuromuscular disease, or immunocompromise [24]. Several economic analyses have not found favorable cost-effectiveness ratios for a variety of these high-risk group definitions, which has resulted in several updates of the AAP guidelines that have narrowed the definition of high-risk groups [25–27]. The availability of long-acting RSV monoclonal antibodies or RSV maternal vaccines that offer protection throughout the entire RSV season with one dose might provide cost-effective alternatives in this regard [28, 29].

Our observed trend of rapidly declining infection rates with age is in concordance with the literature suggesting that RSV infections are most common in infants less than 1 year [16, 19, 30]. Similarly, we observed that most re-infections occurred among children less than 1 year with declining re-infection rates up to age 3. Children aged 4 years had higher re-infection rates relative to those aged 3 years. The observed re-infection rates for this age group reflect unstable estimates from only a few children having an index episode–demonstrated by wide confidence intervals (Tables 1 and 2). For most years, we did not detect any inpatient re-infections in the 4-year age group, owing to increasingly small denominator of the sample with an index infection as well as small re-infection rates.

Although seasonal estimates covered only 4 months, seasonal infection rates were only slightly less than annual estimates, confirming that infections cluster during the RSV season. However, we observed that annual re-infection rates were more than two times higher than seasonal estimates, suggesting that re-infections are more broadly spread across the year. The observation that the risk for RSV re-infection may not be attenuated among children with initial infection paired with the higher susceptibility outside of the core season suggests that re-infections might be focused on certain high-risk groups, although our sample size limited further subgroup analyses [14, 31–33]. Further investigation into the characteristics of children who experience a RSV re-infection in the same year is warranted.

A primary strength of our study is that it includes a large cohort of children, providing population-based evidence on re-infection rates. Furthermore, this is to our knowledge the first epidemiologic study to estimate RSV re-infection rates within the same season. Because we used administrative claims data to derive our estimates, it is possible that multiple RSV-related medical encounters were associated with a single RSV episode. To address this, we developed and applied an algorithm to identify unique RSV episodes using a 30-day washout between individual medical encounters. Sensitivity analyses varying the wash-out periods suggested that our algorithm was robust for estimating annual re-infection rates.

A limitation of our study is that the use of ICD coding on routine healthcare encounters to define RSV episodes could result in underestimation of the true infection rate in the population since not all cases receive a diagnostic RSV test or not all confirmed cases may be specifically coded for RSV in claims data. Studies suggest that about 25% of RSV infections among children less than 5 years do not receive an ICD code for RSV [16]. Importantly, we have previously demonstrated that testing practice varies based on healthcare setting, patient characteristics and the severity of respiratory illness [34]. It is unclear whether history of a recent RSV infection may increase or decrease the propensity to test for RSV when evaluating a re-infection, and thus, direct comparisons between infection and re-infection rates need to consider the impact of testing preferences.

## Conclusion

In summary, the number of medically-attended RSV re-infections within the same season were generally low, particularly those requiring hospitalization. However, re-infections among those with previous infection in the same season was of similar magnitude as the baseline infection risk, suggesting that a previous infection may not attenuate the risk for a re-infection.

## Supporting information

S1 Fig Annual Respiratory Syncytial Virus (RSV) and Respiratory Syncytial Virus Lower Respiratory Tract (RSV LRTI) re-infection rate among commercially-insured children 0–4 years with an index inpatient or outpatient episode in the same year, 2011–2019. **A.** ≥15 days wash out between each episode (RSV), **B.** ≥30 days wash out between each episode (RSV), **C.** ≥45 days wash out between each episode (RSV), **D.** ≥30 days wash out between each episode restricted to RSV LRTI.
(TIF)

S1 Table Annual respiratory syncytial virus infection rate among commercially-insured children 0–4 Years, 2011–2019. (DOCX)

S2 Table Annual inpatient respiratory syncytial virus re-infection rate among commercially-insured children 0–4 years with an index inpatient or outpatient episode in the same year, 2011–2019 –at least 15 days between unique episodes. (DOCX)

S3 Table Annual outpatient respiratory syncytial virus re-infection rate among commercially-insured children 0–4 years with an index inpatient or outpatient episode in the same year, 2011–2019 –at least 15 days between unique episodes. (DOCX)

S4 Table Annual inpatient respiratory syncytial virus re-infection rate among commercially-insured children 0–4 years with an index inpatient or outpatient episode in the same year, 2011–2019 –at least 45 days between unique episodes. (DOCX)

S5 Table Annual outpatient respiratory syncytial virus re-infection rate among commercially-insured children 0–4 years with an index inpatient or outpatient episode in the same year, 2011–2019 –at least 45 days between unique episodes. (DOCX)

S6 Table Annual inpatient respiratory syncytial virus lower respiratory tract re-infection rate among commercially-insured children 0–4 years with an index inpatient or outpatient episode in the same year, 2011–2019. (DOCX)

S7 Table Annual outpatient respiratory syncytial virus lower respiratory tract re-infection rate among commercially-insured children 0–4 years with an index inpatient or outpatient episode in the same year, 2011–2019. (DOCX)
