# Supplementary figures and images for: Respiratory syncytial virus reinfections among infants and young children in the United States, 2011–2019
Source: PLoS One. 2023 Feb 16;18(2):e0281555. doi: 10.1371/journal.pone.0281555 (PMC9934310; doi:10.1371/journal.pone.0281555)

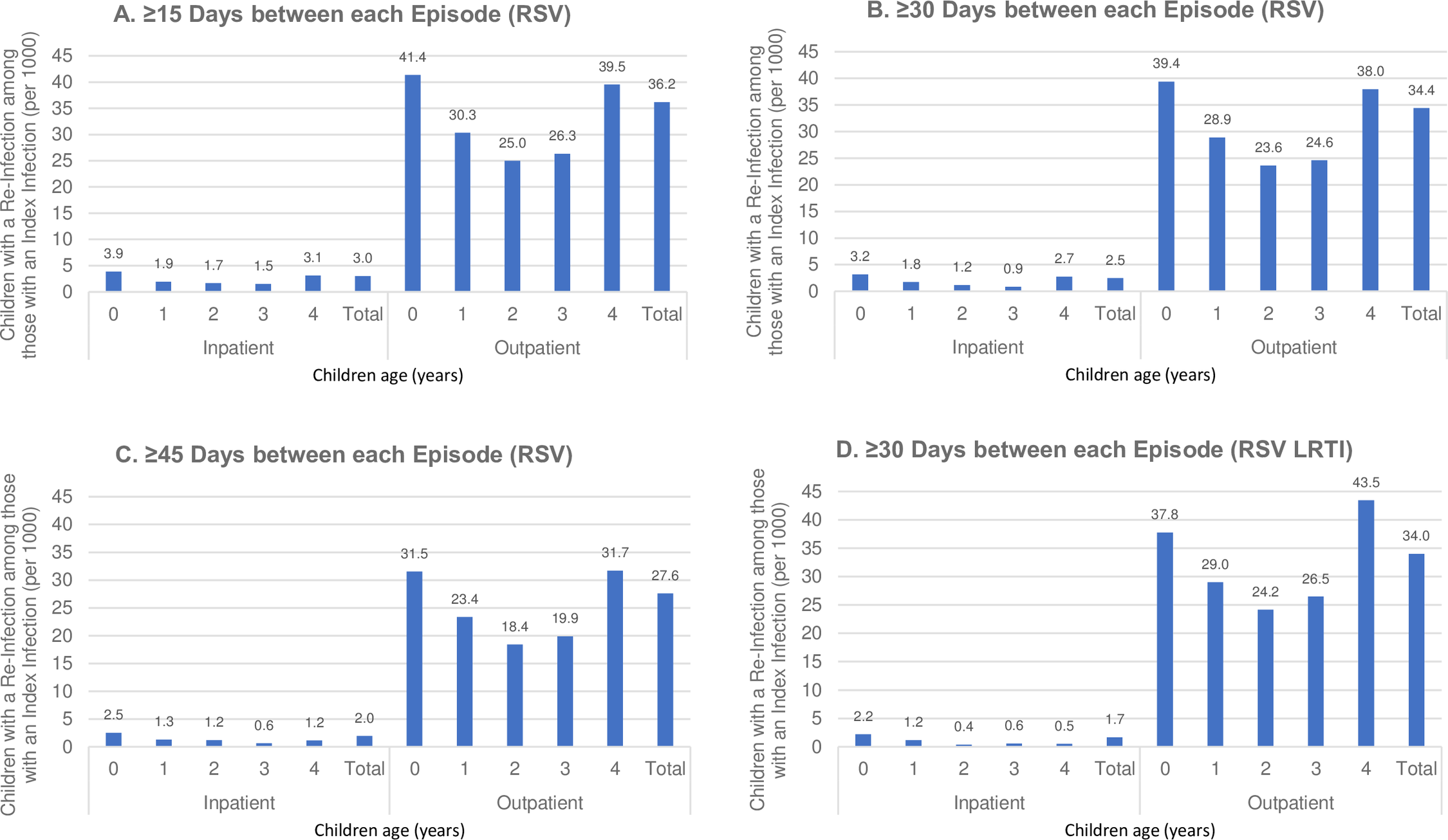

Supplement: S1 Fig — A. ≥15 days wash out between each episode (RSV), B. ≥30 days wash out between each episode (RSV), C. ≥45 days wash out between each episode (RSV), D. ≥30 days wash out between each episode restricted to RSV LRTI. (TIF) [file pone.0281555.s001.tif]
