# Supplementary material for: Respiratory syncytial virus reinfections among infants and young children in the United States, 2011–2019
Source: PLoS One. 2023 Feb 16;18(2):e0281555. doi: 10.1371/journal.pone.0281555 (PMC9934310; doi:10.1371/journal.pone.0281555)
Supplement: S1 Table — (DOCX) [file pone.0281555.s002.docx]

**S1 Table: Annual Respiratory Syncytial Virus Infection Rate among Commercially-Insured Children 0-4 Years, 2011-2019**

|  | Number of Children at Risk  Total Number of Infections  Number of Children with ≥1 Inpatient Episode (%)  Number of Children with ≥1 Outpatient Episode (%) | | | | | |
| --- | --- | --- | --- | --- | --- | --- |
|  | Overall | 0 Years | 1 Year | 2 Years | 3 Years | 4 Years |
| 2011-2012 | 1,110,528  15,348  1,476 (0.13)  13,129 (1.18) | 192,421  8,707  816 (0.42)  7,418 (3.86) | 210,173  3,669  359 (0.17)  3,167 (1.51) | 224,256  1,620  164 (0.07)  1,395 (0.62) | 236,600  813  99 (0.04)  679 (0.29) | 247,078  539  38 (0.02)  470 (0.19) |
| 2012-2013 | 906,333  13,517  1,340 (0.15)  11,615 (1.28) | 158,965  7,758  714 (0.45)  6,666 (4.19) | 172,790  3,315  342 (0.20)  2,862 (1.66) | 181,032  1,379  151 (0.08)  1,190 (0.66) | 190,886  662  92 (0.05)  557 (0.29) | 202,660  403  41 (0.02)  340 (0.17) |
| 2013-2014 | 956,294  12,662  1,112 (0.12)  10,978 (1.15) | 171,024  7,467  627 (0.37)  6,463 (3.78) | 183,203  3,008  241 (0.13)  2,646 (1.44) | 191,706  1,252  139 (0.07)  1,079 (0.56) | 199,999  622  74 (0.04)  529 (0.26) | 210,362  313  31 (0.01)  261 (0.12) |
| 2014-2015 | 803,203  11,852  1,077 (0.13)  10,288 (1.28) | 141,972  6,874  589 (0.41)  5,971 (4.21) | 156,164  2,845  242 (0.15)  2,510 (1.61) | 161,525  1,279  137 (0.08)  1,096 (0.68) | 168,163  547  65 (0.04)  460 (0.27) | 175,379  307  44 (0.03)  251 (0.14) |
| 2015-2016 | 812,404  11,597  1,051 (0.13)  10,202 (1.26) | 145,089  6,562  570 (0.39)  5,766 (3.97) | 158,110  2,923  254 (0.16)  2,590 (1.64) | 164,470  1,242  124 (0.08)  1,096 (0.67) | 169,005  582  77 (0.05)  492 (0.29) | 175,730  288  26 (0.01)  258 (0.15) |
| 2016-2017 | 721,265  11,210  1,006 (0.14)  9,911 (1.37) | 134,204  6,607  571 (0.43)  5,850 (4.36) | 138,405  2,709  241 (0.17)  2,400 (1.73) | 145,071  1,153  108 (0.07)  1,017 (0.70) | 149,990  491  56 (0.04)  430 (0.29) | 153,595  250  30 (0.02)  214 (0.14) |
| 2017-2018 | 687,763  10,672  1,034 (0.15)  9,303 (1.35) | 126,028  6,285  581 (0.46)  5,490 (4.36) | 132,160  2,515  235 (0.18)  2,206 (1.67) | 138,360  1,085  120 (0.09)  944 (0.68) | 143,561  504  64 (0.04)  429 (0.30) | 147,654  283  34 (0.02)  234 (0.16) |
| 2018-2019 | 708,189  12,506  1,195 (0.17)  10,945 (1.55) | 129,447  7,337  679 (0.52)  6,417 (4.96) | 134,897  2,946  276 (0.20)  2,595 (1.92) | 142,558  1,320  137 (0.10)  1,161 (0.81) | 148,375  600  56 (0.04)  526 (0.35) | 152,912  303  47 (0.03)  246 (0.16) |
| Total | 6,705,979  99,364  9,291 (0.14)  86,371 (1.29) | 1,199,150  57,597  5,147 (0.43)  50,041 (4.17) | 1,285,902  23,930  2,190 (0.17)  20,976 (1.63) | 1,348,978  10,330  1,080 (0.08)  8,978 (0.67) | 1,406,579  4,821  583 (0.04)  4,102 (0.29) | 1,465,370  2,686  291 (0.02)  2,274 (0.16) |
