# Supplementary material for: Respiratory syncytial virus reinfections among infants and young children in the United States, 2011–2019
Source: PLoS One. 2023 Feb 16;18(2):e0281555. doi: 10.1371/journal.pone.0281555 (PMC9934310; doi:10.1371/journal.pone.0281555)
Supplement: S2 Table — (DOCX) [file pone.0281555.s003.docx]

**S2 Table: Annual Inpatient Respiratory Syncytial Virus Re-Infection Rate among Commercially-Insured Children 0-4 Years with an Index Inpatient or Outpatient Episode in the Same Year, 2011-2019 – At Least 15 Days between Unique Episodes**^a^

|  | Children with Index Episode in either Inpatient or Outpatient Setting (N)  Number of Inpatient Re-infections  Children with ≥1 Inpatient Re-infection (N)  Inpatient Re-infection Rate, % (95% Confidence Interval) | | | | | |
| --- | --- | --- | --- | --- | --- | --- |
|  | Overall | 0 Years | 1 Year | 2 Years | 3 Years | 4 Years |
| 2011-2012 | 14,533  56  56  0.39 (0.28-0.49) | 8,181  42  42  0.51 (0.36-0.67) | 3,515  9  9  0.26 (0.09-0.42) | 1,555  4  4  0.26 (0.01-0.51) | 774  1  1  0.13 (0.00-0.38)^b^ | 508  0  0  0.00 (0.00-0.00) |
| 2012-2013 | 12,898  37  36  0.28 (0.19-0.37) | 7,342  23  22  0.30 (0.17-0.42) | 3,190  11  11  0.34 (0.14-0.55) | 1,340  1  1  0.07 (0.00-0.22)^b^ | 647  1  1  0.15 (0.00-0.46)^b^ | 379  1  1  0.26 (0.00-0.78)^b^ |
| 2013-2014 | 12,033  37  36  0.30 (0.20-0.40) | 7,051  30  29  0.41 (0.26-0.56) | 2,873  4  4  0.14 (0.00-0.28) | 1,216  3  3  0.25 (0.00-0.53)^b^ | 602  0  0  0.00 (0.00-0.00) | 291  0  0  0.00 (0.00-0.00) |
| 2014-2015 | 11,317  28  26  0.23 (0.14-0.32) | 6,522  24  22  0.34 (0.20-0.48) | 2,747  0  0  0.00 (0.00-0.00) | 1,231  1  1  0.08 (0.00-0.24)^b^ | 524  1  1  0.19 (0.00-0.56)^b^ | 293  2  2  0.68 (0.00-1.63)^b^ |
| 2015-2016 | 11,211  32  31  0.28 (0.18-0.37) | 6,306  25  24  0.38 (0.23-0.53) | 2,840  5  5  0.18 (0.02-0.33) | 1,216  1  1  0.08 (0.00-0.24)^b^ | 568  1  1  0.18 (0.00-0.52)^b^ | 281  0  0  0.00 (0.00-0.00) |
| 2016-2017 | 10,883  33  32  0.29 (0.19-0.40) | 6,396  27  26  0.41 (0.25-0.56) | 2,637  3  3  0.11 (0.00-0.24)^b^ | 1,122  2  2  0.18 (0.00-0.43)^b^ | 484  1  1  0.21 (0.00-0.61)^b^ | 244  0  0  0.00 (0.00-0.00) |
| 2017-2018 | 10,296  37  36  0.35 (0.24-0.46) | 6,044  22  22  0.36 (0.21-0.52) | 2,434  6  6  0.25 (0.05-0.44) | 1,060  5  5  0.47 (0.06-0.88) | 492  0  0  0.00 (0.00-0.00) | 266  4  3  1.13 (0.00-2.40)^b^ |
| 2018-2019 | 12,098  37  36  0.30 (0.20-0.39) | 7,063  25  25  0.35 (0.22-0.49) | 2,863  8  7  0.24 (0.06-0.43) | 1,298  0  0  0.00 (0.00-0.00) | 581  2  2  0.34 (0.00-0.82)^b^ | 293  2  2  0.68 (0.00-1.63)^b^ |
| Total | 95,269  297  289  0.30 (0.27-0.34) | 54,905  218  212  0.39 (0.33-0.44) | 23,099  46  45  0.19 (0.14-0.25) | 10,038  17  17  0.17 (0.09-0.25) | 4,672  7  7  0.15 (0.04-0.26) | 2,555  9  8  0.31 (0.10-0.53) |

^a^Index episode may occur in either the inpatient or outpatient setting

^b^Negative 95% confidence limit truncated to 0.00%
