# Supplementary material for: Respiratory syncytial virus reinfections among infants and young children in the United States, 2011–2019
Source: PLoS One. 2023 Feb 16;18(2):e0281555. doi: 10.1371/journal.pone.0281555 (PMC9934310; doi:10.1371/journal.pone.0281555)
Supplement: S3 Table — (DOCX) [file pone.0281555.s004.docx]

**S3 Table: Annual Outpatient Respiratory Syncytial Virus Re-Infection Rate among Commercially-Insured Children 0-4 Years with an Index Inpatient or Outpatient Episode in the Same Year, 2011-2019 – At Least 15 Days between Unique Episodes**^a^

|  | Children with Index Episode in either Inpatient or Outpatient Setting (N)  Number of Outpatient Re-infections  Children with ≥1 Outpatient Re-infection (N)  Outpatient Re-infection Rate (95% Confidence Interval) | | | | | |
| --- | --- | --- | --- | --- | --- | --- |
|  | Overall | 0 Years | 1 Year | 2 Years | 3 Years | 4 Years |
| 2011-2012 | 14,533  787  664  4.57 (4.23-4.91) | 8,181  503  430  5.26 (4.77-5.74) | 3,515  148  124  3.53 (2.92-4.14) | 1,555  65  52  3.34 (2.45-4.24) | 774  39  32  4.13 (2.73-5.54) | 508  32  26  5.12 (3.20-7.03) |
| 2012-2013 | 12,898  609  520  4.03 (3.69-4.37) | 7,342  407  351  4.78 (4.29-5.27) | 3,190  122  101  3.17 (2.56-3.77) | 1,340  41  35  2.61 (1.76-3.47) | 647  14  14  2.16 (1.04-3.28) | 379  25  19  5.01 (2.82-7.21) |
| 2013-2014 | 12,033  618  532  4.42 (4.05-4.79) | 7,051  403  341  4.84 (4.34-5.34) | 2,873  135  119  4.14 (3.41-4.87) | 1,216  36  35  2.88 (1.94-3.82) | 602  22  20  3.32 (1.89-4.75) | 291  22  17  5.84 (3.15-8.54) |
| 2014-2015 | 11,317  534  457  4.04 (3.68-4.40) | 6,522  346  298  4.57 (4.06-5.08) | 2,747  103  90  3.28 (2.61-3.94) | 1,231  49  43  3.49 (2.47-4.52) | 524  23  15  2.86 (1.43-4.29) | 293  13  11  3.75 (1.58-5.93) |
| 2015-2016 | 11,211  382  337  3.01 (2.69-3.32) | 6,306  252  223  3.54 (3.08-3.99) | 2,840  82  74  2.61 (2.02-3.19) | 1,216  26  23  1.89 (1.13-2.66) | 568  15  11  1.94 (0.80-3.07) | 281  7  6  2.14 (0.45-3.83) |
| 2016-2017 | 10,883  323  280  2.57 (2.28-2.87) | 6,396  207  180  2.81 (2.41-3.22) | 2,637  73  60  2.28 (1.71-2.84) | 1,122  30  27  2.41 (1.51-3.30) | 484  7  7  1.45 (0.38-2.51) | 244  6  6  2.46 (0.52-4.40) |
| 2017-2018 | 10,296  374  324  3.15 (2.81-3.48) | 6,044  241  210  3.47 (3.01-3.94) | 2,434  82  73  3.00 (2.32-3.68) | 1,060  25  20  1.89 (1.07-2.71) | 492  12  9  1.83 (0.65-3.01) | 266  14  12  4.51 (2.02-7.01) |
| 2018-2019 | 12,098  400  333  2.75 (2.46-3.04) | 7,063  268  238  3.37 (2.95-3.79) | 2,863  80  60  2.10 (1.57-2.62) | 1,298  23  16  1.23 (0.63-1.83) | 581  21  15  2.58 (1.29-3.87) | 293  8  4  1.37 (0.04-2.69) |
| Total | 95,269  4,027  3,447  3.62 (3.50-3.74) | 54,905  2,627  2,271  4.14 (3.97-4.30) | 23,099  825  701  3.03 (2.81-3.26) | 10,038  295  251  2.5 (2.20-2.81) | 4,672  153  123  2.63 (2.17-3.09) | 2,555  127  101  3.95 (3.20-4.71) |

^a^Index episode may occur in either the inpatient or outpatient setting
