# Supplementary material for: Respiratory syncytial virus reinfections among infants and young children in the United States, 2011–2019
Source: PLoS One. 2023 Feb 16;18(2):e0281555. doi: 10.1371/journal.pone.0281555 (PMC9934310; doi:10.1371/journal.pone.0281555)
Supplement: S4 Table — (DOCX) [file pone.0281555.s005.docx]

**S4 Table: Annual Inpatient Respiratory Syncytial Virus Re-Infection Rate among Commercially-Insured Children 0-4 Years with an Index Inpatient or Outpatient Episode in the Same Year, 2011-2019 – At Least 45 Days between Unique Episodes**^a^

|  | Children with Index Episode in either Inpatient or Outpatient Setting (N)  Number of Inpatient Re-infections  Children with ≥1 Inpatient Re-infection (N)  Inpatient Re-infection Rate, % (95% Confidence Interval) | | | | | |
| --- | --- | --- | --- | --- | --- | --- |
|  | Overall | 0 Years | 1 Year | 2 Years | 3 Years | 4 Years |
| 2011-2012 | 14,533  37  37  0.25 (0.17-0.34) | 8,181  26  26  0.32 (0.20-0.44) | 3,515  8  8  0.23 (0.07-0.39) | 1,555  3  3  0.19 (0.00-0.41)^b^ | 774  0  0  0.00 (0.00-0.00) | 508  0  0  0.00 (0.00-0.00) |
| 2012-2013 | 12,898  22  22  0.17 (0.10-0.24) | 7,342  12  12  0.16 (0.07-0.26) | 3,190  8  8  0.25 (0.08-0.42) | 1,340  0  0  0.00 (0.00-0.00) | 647  1  1  0.15 (0.00-0.46)^b^ | 379  1  1  0.26 (0.00-0.78)^b^ |
| 2013-2014 | 12,033  27  26  0.22 (0.13-0.30) | 7,051  22  21  0.30 (0.17-0.43) | 2,873  3  3  0.10 (0.00-0.22)^b^ | 1,216  2  2  0.16 (0.00-0.39)^b^ | 602  0  0  0.00 (0.00-0.00) | 291  0  0  0.00 (0.00-0.00) |
| 2014-2015 | 11,317  20  19  0.17 (0.09-0.24) | 6,522  18  17  0.26 (0.14-0.38) | 2,747  0  0  0.00 (0.00-0.00) | 1,231  1  1  0.08 (0.00-0.24)^b^ | 524  1  1  0.19 (0.00-0.56)^b^ | 293  0  0  0.00 (0.00-0.00) |
| 2015-2016 | 11,211  24  24  0.21 (0.13-0.30) | 6,306  18  18  0.29 (0.15-0.42) | 2,840  4  4  0.14 (0.00-0.28) | 1,216  1  1  0.08 (0.00-0.24)^b^ | 568  1  1  0.18 (0.00-0.52)^b^ | 281  0  0  0.00 (0.00-0.00) |
| 2016-2017 | 10,883  15  14  0.13 (0.06-0.20) | 6,396  12  11  0.17 (0.07-0.27) | 2,637  1  1  0.04 (0.00-0.11)^b^ | 1,122  2  2  0.18 (0.00-0.43)^b^ | 484  0  0  0.00 (0.00-0.00) | 244  0  0  0.00 (0.00-0.00) |
| 2017-2018 | 10,296  22  22  0.21 (0.12-0.30) | 6,044  15  15  0.25 (0.12-0.37) | 2,434  3  3  0.12 (0.00-0.26)^b^ | 1,060  3  3  0.28 (0.00-0.60)^b^ | 492  0  0  0.00 (0.00-0.00) | 266  1  1  0.38 (0.00-1.11)^b^ |
| 2018-2019 | 12,098  22  22  0.18 (0.11-0.26) | 7,063  18  18  0.25 (0.14-0.37) | 2,863  3  3  0.10 (0.00-0.22)^b^ | 1,298  0  0  0.00 (0.00-0.00) | 581  0  0  0.00 (0.00-0.00) | 293  1  1  0.34 (0.00-1.01)^b^ |
| Total | 95,269  189  186  0.2 (0.17-0.22) | 54,905  141  138  0.25 (0.21-0.29) | 23,099  30  30  0.13 (0.08-0.18) | 10,038  12  12  0.12 (0.05-0.19) | 4,672  3  3  0.06 (0.00-0.14)^b^ | 2,555  3  3  0.12 (0.00-0.25)^b^ |

^a^Index episode may occur in either the inpatient or outpatient setting

^b^Negative 95% confidence limit truncated to 0.00%
