# Supplementary material for: Respiratory syncytial virus reinfections among infants and young children in the United States, 2011–2019
Source: PLoS One. 2023 Feb 16;18(2):e0281555. doi: 10.1371/journal.pone.0281555 (PMC9934310; doi:10.1371/journal.pone.0281555)
Supplement: S5 Table — (DOCX) [file pone.0281555.s006.docx]

**S5 Table: Annual Outpatient Respiratory Syncytial Virus Re-Infection Rate among Commercially-Insured Children 0-4 Years with an Index Inpatient or Outpatient Episode in the Same Year, 2011-2019 – At Least 45 Days between Unique Episodes**^a^

|  | Children with Index Episode in either Inpatient or Outpatient Setting (N)  Number of Outpatient Re-infections  Children with ≥1 Outpatient Re-infection (N)  Outpatient Re-infection Rate (95% Confidence Interval) | | | | | |
| --- | --- | --- | --- | --- | --- | --- |
|  | Overall | 0 Years | 1 Year | 2 Years | 3 Years | 4 Years |
| 2011-2012 | 14,533  602  543  3.74 (3.43-4.04) | 8,181  385  347  4.24 (3.80-4.68) | 3,515  116  103  2.93 (2.37-3.49) | 1,555  44  43  2.77 (1.95-3.58) | 774  32  28  3.62 (2.30-4.93) | 508  25  22  4.33 (2.56-6.10) |
| 2012-2013 | 12,898  441  401  3.11 (2.81-3.41) | 7,342  301  275  3.75 (3.31-4.18) | 3,190  81  74  2.32 (1.80-2.84) | 1,340  26  24  1.79 (1.08-2.50) | 647  11  11  1.70 (0.70-2.70) | 379  22  17  4.49 (2.40-6.57) |
| 2013-2014 | 12,033  457  419  3.48 (3.15-3.81) | 7,051  298  273  3.87 (3.42-4.32) | 2,873  98  91  3.17 (2.53-3.81) | 1,216  23  23  1.89 (1.13-2.66) | 602  19  17  2.82 (1.50-4.15) | 291  19  15  5.15 (2.61-7.70) |
| 2014-2015 | 11,317  375  340  3.00 (2.69-3.32) | 6,522  236  216  3.31 (2.88-3.75) | 2,747  77  69  2.51 (1.93-3.10) | 1,231  41  37  3.01 (2.05-3.96) | 524  12  10  1.91 (0.74-3.08) | 293  9  8  2.73 (0.86-4.60) |
| 2015-2016 | 11,211  263  245  2.19 (1.91-2.46) | 6,306  180  167  2.65 (2.25-3.04) | 2,840  56  53  1.87 (1.37-2.36) | 1,216  18  16  1.32 (0.68-1.96) | 568  5  5  0.88 (0.11-1.65) | 281  4  4  1.42 (0.04-2.81) |
| 2016-2017 | 10,883  222  205  1.88 (1.63-2.14) | 6,396  136  127  1.99 (1.64-2.33) | 2,637  52  46  1.74 (1.24-2.24) | 1,122  22  20  1.78 (1.01-2.56) | 484  7  7  1.45 (0.38-2.51) | 244  5  5  2.05 (0.27-3.83) |
| 2017-2018 | 10,296  238  224  2.18 (1.89-2.46) | 6,044  151  143  2.37 (1.98-2.75) | 2,434  59  55  2.26 (1.67-2.85) | 1,060  14  12  1.13 (0.50-1.77) | 492  6  6  1.22 (0.25-2.19) | 266  8  8  3.01 (0.95-5.06) |
| 2018-2019 | 12,098  270  253  2.09 (1.84-2.35) | 7,063  192  183  2.59 (2.22-2.96) | 2,863  54  49  1.71 (1.24-2.19) | 1,298  11  10  0.77 (0.29-1.25) | 581  10  9  1.55 (0.54-2.55) | 293  3  2  0.68 (0.00-1.63)^b^ |
| Total | 95,269  2,868  2,630  2.76 (2.66-2.86) | 54,905  1,879  1,731  3.15 (3.01-3.30) | 23,099  593  540  2.34 (2.14-2.53) | 10,038  199  185  1.84 (1.58-2.11) | 4,672  102  93  1.99 (1.59-2.39) | 2,555  95  81  3.17 (2.49-3.85) |

^a^Index episode may be occur in either the inpatient or outpatient setting
