# Supplementary material for: Respiratory syncytial virus reinfections among infants and young children in the United States, 2011–2019
Source: PLoS One. 2023 Feb 16;18(2):e0281555. doi: 10.1371/journal.pone.0281555 (PMC9934310; doi:10.1371/journal.pone.0281555)
Supplement: S6 Table — (DOCX) [file pone.0281555.s007.docx]

**S6 Table: Annual Inpatient Respiratory Syncytial Virus Lower Respiratory Tract Re-Infection Rate among Commercially-Insured Children 0-4 Years with an Index Inpatient or Outpatient Episode in the Same Year, 2011-2019**^a^

|  | Children with Index Lower Respiratory Tract RSV Infection Episode in either Inpatient or Outpatient Setting (N)  Number of Inpatient Lower Respiratory Tract RSV Re-infections  Children with ≥1 Lower Respiratory Tract RSV Inpatient Re-infection (N)  Inpatient Lower Respiratory Tract RSV Re-infection Rate, % (95% Confidence Interval) | | | | | |
| --- | --- | --- | --- | --- | --- | --- |
|  | Overall | 0 Years | 1 Year | 2 Years | 3 Years | 4 Years |
| 2011-2012 | 11,855  28  28  0.24 (0.15-0.32) | 6,788  24  24  0.35 (0.21-0.49) | 2,791  4  4  0.14 (0.00-0.28) | 1,224  0  0  0.00 (0.00-0.00) | 624  0  0  0.00 (0.00-0.00) | 428  0  0  0.00 (0.00-0.00) |
| 2012-2013 | 10,187  15  15  0.15 (0.07-0.22) | 5,951  9  9  0.15 (0.05-0.25) | 2,452  6  6  0.24 (0.05-0.44) | 988  0  0  0.00 (0.00-0.00) | 505  0  0  0.00 (0.00-0.00) | 291  0  0  0.00 (0.00-0.00) |
| 2013-2014 | 9,412  15  15  0.16 (0.08-0.24) | 5,646  12  12  0.21 (0.09-0.33) | 2,171  3  3  0.14 (0.00-0.29)^b^ | 923  0  0  0.00 (0.00-0.00) | 455  0  0  0.00 (0.00-0.00) | 217  0  0  0.00 (0.00-0.00) |
| 2014-2015 | 8,583  16  15  0.17 (0.09-0.26) | 5,120  14  13  0.25 (0.12-0.39) | 2,015  0  0  0.00 (0.00-0.00) | 875  1  1  0.11 (0.00-0.34)^b^ | 373  1  1  0.27 (0.00-0.79)^b^ | 200  0  0  0.00 (0.00-0.00) |
| 2015-2016 | 9,074  14  14  0.15 (0.07-0.24) | 5,278  10  10  0.19 (0.07-0.31) | 2,260  2  2  0.09 (0.00-0.21)^b^ | 917  1  1  0.11 (0.00-0.32)^b^ | 411  1  1  0.24 (0.00-0.72)^b^ | 208  0  0  0.00 (0.00-0.00) |
| 2016-2017 | 8,606  10  10  0.12 (0.04-0.19) | 5,269  10  10  0.19 (0.07-0.31) | 2,014  0  0  0.00 (0.00-0.00) | 821  0  0  0.00 (0.00-0.00) | 349  0  0  0.00 (0.00-0.00) | 153  0  0  0.00 (0.00-0.00) |
| 2017-2018 | 8,172  11  11  0.13 (0.06-0.21) | 5,015  9  9  0.18 (0.06-0.30) | 1,883  1  1  0.05 (0.00-0.16)^b^ | 779  1  1  0.13 (0.00-0.38)^b^ | 317  0  0  0.00 (0.00-0.00) | 178  0  0  0.00 (0.00-0.00) |
| 2018-2019 | 9,567  19  19  0.20 (0.11-0.29) | 5,885  13  13  0.22 (0.10-0.34) | 2,166  5  5  0.23 (0.03-0.43) | 923  0  0  0.00 (0.00-0.00) | 404  0  0  0.00 (0.00-0.00) | 189  1  1  0.53 (0.00-1.56)^b^ |
| Total | 75,456  128  127  0.17 (0.14-0.20) | 44,952  101  100  0.22 (0.18-0.27) | 17,752  21  21  0.12 (0.07-0.17) | 7,450  3  3  0.04 (0.00-0.09)^b^ | 3,438  2  2  0.06 (0.00-0.14)^b^ | 1,864  1  1  0.05 (0.00-0.16)^b^ |

^a^Index episode may be occur in either the inpatient or outpatient setting

^b^Negative 95% confidence limit truncated to 0.00%
