# Supplementary material for: Respiratory syncytial virus reinfections among infants and young children in the United States, 2011–2019
Source: PLoS One. 2023 Feb 16;18(2):e0281555. doi: 10.1371/journal.pone.0281555 (PMC9934310; doi:10.1371/journal.pone.0281555)
Supplement: S7 Table — (DOCX) [file pone.0281555.s008.docx]

**S7 Table: Annual Outpatient Respiratory Syncytial Virus Lower Respiratory Tract Re-Infection Rate among Commercially-Insured Children 0-4 Years with an Index Inpatient or Outpatient Episode in the Same Year, 2011-2019^a^**

|  | Children with Index Episode in either Inpatient or Outpatient Setting (N)  Number of Outpatient Re-infections  Children with ≥1 Outpatient Re-infection (N)  Outpatient Re-infection Rate (95% Confidence Interval) | | | | | |
| --- | --- | --- | --- | --- | --- | --- |
|  | Overall | 0 Years | 1 Year | 2 Years | 3 Years | 4 Years |
| 2011-2012 | 11,855  656  555  4.68 (4.30-5.06) | 6,788  407  353  5.20 (4.67-5.73) | 2,791  126  105  3.76 (3.06-4.47) | 1,224  58  45  3.68 (2.62-4.73) | 624  34  27  4.33 (2.73-5.92) | 428  31  25  5.84 (3.62-8.06) |
| 2012-2013 | 10,187  482  409  4.01 (3.63-4.40) | 5,951  322  277  4.65 (4.12-5.19) | 2,452  97  79  3.22 (2.52-3.92) | 988  31  27  2.73 (1.72-3.75) | 505  12  12  2.38 (1.05-3.70) | 291  20  14  4.81 (2.35-7.27) |
| 2013-2014 | 9,412  465  400  4.25 (3.84-4.66) | 5,646  306  259  4.59 (4.04-5.13) | 2,171  99  89  4.10 (3.27-4.93) | 923  25  24  2.60 (1.57-3.63) | 455  16  14  3.08 (1.49-4.66) | 217  19  14  6.45 (3.18-9.72) |
| 2014-2015 | 8,583  379  322  3.75 (3.35-4.15) | 5,120  247  213  4.16 (3.61-4.71) | 2,015  68  61  3.03 (2.28-3.78) | 875  34  28  3.20 (2.03-4.37) | 373  20  12  3.22 (1.43-5.01) | 200  10  8  4.00 (1.28-6.72) |
| 2015-2016 | 9,074  281  244  2.69 (2.36-3.02) | 5,278  184  161  3.05 (2.59-3.51) | 2,260  62  56  2.48 (1.84-3.12) | 917  19  16  1.74 (0.90-2.59) | 411  11  7  1.70 (0.45-2.95) | 208  5  4  1.92 (0.06-3.79) |
| 2016-2017 | 8,606  232  197  2.29 (1.97-2.61) | 5,269  153  129  2.45 (2.03-2.87) | 2,014  53  43  2.14 (1.50-2.77) | 821  18  17  2.07 (1.10-3.04) | 349  4  4  1.15 (0.03-2.26) | 153  4  4  2.61 (0.09-5.14) |
| 2017-2018 | 8,172  248  207  2.53 (2.19-2.87) | 5,015  156  133  2.65 (2.21-3.1) | 1,883  54  46  2.44 (1.75-3.14) | 779  18  13  1.67 (0.77-2.57) | 317  9  6  1.89 (0.39-3.39) | 178  11  9  5.06 (1.84-8.27) |
| 2018-2019 | 9,567  284  231  2.41 (2.11-2.72) | 5,885  196  173  2.94 (2.51-3.37) | 2,166  51  36  1.66 (1.12-2.20) | 923  16  10  1.08 (0.42-1.75) | 404  14  9  2.23 (0.79-3.67) | 189  7  3  1.59 (0.00-3.37)^b^ |
| Total | 75,456  3,027  2,565  3.40 (3.27-3.53) | 44,952  1,971  1,698  3.78 (3.60-3.95) | 17,752  610  515  2.90 (2.65-3.15) | 7,450  219  180  2.42 (2.07-2.76) | 3,438  120  91  2.65 (2.11-3.18) | 1,864  107  81  4.35 (3.42-5.27) |

^a^Index episode may be occur in either the inpatient or outpatient setting

^b^Negative 95% confidence limit truncated to 0.00%
